# Development of Avocado and Lemon Oil Emulgels Based on Natural Products: Phycocyanin and Pectin

**DOI:** 10.3390/pharmaceutics15082067

**Published:** 2023-08-01

**Authors:** Patricia Tello, Nuria Calero, Jenifer Santos, Luis A. Trujillo-Cayado

**Affiliations:** 1 Departamento de Ingeniería Química, Escuela Politécnica Superior, Universidad de Sevilla, c/Virgen de África 7, E41011 Sevilla, Spain; pattelriv@alum.us.es (P.T.); ltrujillo@us.es (L.A.T.-C.); 2 Departamento de Ingeniería Química, Facultad de Química, Universidad de Sevilla, c/Profesor García González S/N, E41012 Sevilla, Spain; 3 Facultad de Ciencias de la Salud, Universidad Loyola Andalucía, Avda. de las Universidades s/n, Dos Hermanas, E41703 Sevilla, Spain

**Keywords:** emulgels, microfluidization, pectin, phycocyanin

## Abstract

Phycocyanin (PC), a natural product obtained from algae, is attracting attention due to its health benefits, such as its antioxidant and anti-inflammatory properties. This work studies the use of PC as the main stabilizer in avocado and lemon oil emulgels, a format for drug delivery. The influence of PC concentration on droplet size distribution, rheological properties, and physical stability is studied using a laser diffraction technique, rheological measurements, and multiple light scattering. The 5 wt.% PC emulsions show the lowest droplet size and, consequently, the best stability against creaming and droplet growth. Emulsions formulated with PC as the only stabilizer show a slight pseudoplastic character with an apparent viscosity below 10 mPa·s at 2 Pa. This indicates that these emulsions undergo creaming with aging time. In order to reduce creaming, pectin is incorporated into the 5 wt.% PC emulsion at different concentrations. Interestingly, yield stress and an incipient gel character are observed due to the presence of pectin. This is why the creaming mechanism is reduced. In conclusion, PC forms a layer that protects the interface against coalescence and Ostwald ripening. And, pectin is incorporated to reduce creaming. This research has the potential to make valuable contributions to diverse fields, such as health, medicine, and encapsulation technology.

## 1. Introduction

A sub-micron emulgel format is the combination of an emulsion in the sub-micron scale and a gel base. This dual character makes it an appropriate candidate for the delivery of hydrophobic drugs. On the one hand, the emulsion component of an emulgel protects the active ingredient by reducing some degradation reactions, such as hydrolysis. On the other hand, the gel format increases the viscosity of the aqueous phase, provoking a decrease in the interfacial and surface tension. In addition, emulgels present rheological characteristics that are suitable for topical delivery [1]. As an emerging transdermal delivery tool, emulgel has been proven to show surprising upshots for lipophilic drugs compared to other formulations, with potential applications in the pharmaceutical industry. The lipophilic nature of the majority of newer drugs developed in the modern era results in poor oral bioavailability, erratic absorption, and pharmacokinetic variations. Therefore, this novel transdermal delivery system has proven to be advantageous over other oral and topical drug delivery systems in avoiding such disturbances [2].

In the last decade, food researchers have paid great attention to “Green Chemistry”, which aims at the development of new formulations using renewable resources based on vegetables and algae in terms of the lowest possible impact on human life and the environment. Generally, several compounds, including those from animals, are used for the production of food products, and in some cases, very high-energy input is required. Proteins obtained from animals can contribute to environmental impact. Furthermore, there are studies that recommend a reduction in consuming animal-based products [3]. Therefore, to develop a green approach, researchers have the opportunity to investigate proteins obtained from vegetables or algae to stabilize food products.

Phycocyanin (PC) is generally recognized as a safe (GRAS) pigment found in certain types of blue-green algae, also known as cyanobacteria. It is a protein complex that plays a crucial role in the process of photosynthesis. The name “*phycocyanin*” is derived from the Greek words “*phykos*”, meaning “algae”, and “*kyanos*”, meaning “blue”, reflecting its characteristic blue color. Phycocyanin has significant nutraceutical and pharmaceutical potential based on various studies in recent years. Its anti-inflammatory and antioxidant activities have been clearly demonstrated in food innovation. Due to its potential health benefits, PC has attracted significant attention in various fields, including medicine, food, and cosmetics [4]. Studies have suggested that PC may have anticancer, neuroprotective, and immunomodulatory effects, although further research is still needed to fully understand its mechanisms of action [5]. In the food industry, PC is utilized as a natural food colorant, serving as an alternative to synthetic dyes. Its safety profile and attractive hue make it an appealing choice for various food and beverage products. In addition, PC presents surface activity and could reduce interfacial tension [5]. In addition, its potential as a stabilizing and emulsifying agent has already been tested in some studies, although its applications for the design of encapsulation systems still need to be explored [6,7].

Pectins are complex polysaccharides widely found in plant cell walls. Chemically, pectins are composed of chains of sugar molecules, primarily galacturonic acid. These chains can vary in length and branching patterns, leading to different types of pectins with varying properties. Other sugar molecules, such as rhamnose and arabinose, further add to the complexity of pectin structures. One of the notable characteristics of pectins is their ability to form gels when they interact with water and sugar under specific conditions [8]. This property has made pectins invaluable in the food industry for thickening, stabilizing, and gelling applications. They are commonly used in the production of jams, jellies, fruit preserves, and other food products [9]. Beyond their food uses, pectins have gained attention for their potential health benefits. They are classified as dietary fibers and can contribute to digestive health by promoting regular bowel movements and feeling full [10]. Pectins can also bind to cholesterol, which may help to reduce blood cholesterol levels.

The selection of the oil phase and its relative quantity will depend on the end use of the product. For pharmaceutical, cosmetic, food, and agrochemical applications, oils derived from plants, the so-called essential oils, are a good choice because of their antimicrobial, antifungal, and medicinal properties. Avocado oil, derived from the fruit of the avocado tree (*Persea americana*), has gained considerable popularity in recent years due to its numerous health benefits. The oil is extracted from the fleshy pulp of ripe avocados, resulting in a smooth, greenish-yellow liquid with a distinct aroma and flavor. Avocado oil is highly regarded for its high content of monounsaturated fats, specifically oleic acid, which is known to promote heart health by reducing bad cholesterol levels [11]. Additionally, it contains essential fatty acids, vitamins, and antioxidants that contribute to overall well-being. Because of these properties, avocado oil has gained attention in the last few years for the food industry. Among essential oils, lemon essential oil has properties of great interest for its applications in fields such as cosmetics, food, and medicine. It has beneficial health effects by lowering cholesterol and triglycerides without affecting other blood biochemical parameters [12].

The objective of this study was to develop stable emulsions based on plant-based compounds, with PC as the main emulsifier and avocado and lemon oils as dispersed phases. Furthermore, the combination of PC with a plant-based thickener (pectin) to improve the physical stability of these systems was studied. The influence of pectin concentration on rheological properties and physical stability of emulsions based on phycocyanin was studied. This work sets the stage for future studies based on PC and on a combination of pectin-PC and has the potential to make a valuable contribution to fields as diverse as health, medicine, and encapsulation technology.

## 2. Materials and Methods

### 2.1. Materials

Phycocyanin (PC) was purchased from Ecospirulina (Valencia, Spain) and was used as an emulsifier. The avocado oil and lemon essential oil used as dispersed phases were purchased from Bidah Chaumel (Murcia, Spain) and used as received. Pectin from citrus peel was purchased from Sigma-Aldrich and supplied by Quimidroga (Barcelona, Spain). All compounds were natural products based on a certificate of analysis.

### 2.2. Emulgel Preparation

The aqueous phase contained deionized water at pH 2.5, 0.1 wt.% antifoam agent (Antifoam B Emulsion, Sigma-Aldrich, Saint Louis, MO, USA), and different PC concentrations (1–5 wt.%). The oil phase (20 wt.%) consisted of a mixture of two food-grade oils: avocado oil and lemon oil with 90/10 mass ratio. A three-step procedure was followed to prepare the emulgels. Firstly, a pre-emulsion (150 g) was prepared by primary homogenization using an Ultraturrax T50 (Ika, Staufen, Germany) rotor–stator device at 6000 rpm for 60 s. Subsequently, this pre-emulsion was introduced into a microfluidizer (Model M-110P, Microfluidics, Westwood, MA, USA) operating at 25,000 psi (1724 bar) for one cycle. Finally, various concentrations of citrus peel pectin were added to the emulsion with the optimum concentration of PC (2.5, 5, or 7.5 wt.%). The incorporation of citrus peel pectin was performed by solubilizing the thickener directly in the emulsion with the aid of mechanical stirring at 600 rpm for 5 min. The samples were prepared in triplicate, stored at room temperature, and protected from light in transparent glass vials.

### 2.3. Droplet Size Distribution and Mean Diameter Determination

The droplet size distributions and mean diameters of emulgel oil droplets were measured using a laser diffraction technique (Mastersizer 2000, Malvern, UK). The mean droplet diameters were expressed as volume mean diameter (D_4,3_) and Sauter mean diameter (D_3,2_). In order to quantify the polydispersity, the span parameter was used. All measurements were carried out in triplicate for each sample. The influence of aging time on droplet size distributions was studied for 36 days.

### 2.4. Rheological Characterization

Rheological measurements were carried out using a controlled-stress Haake MARS II rheometer (Thermo-Scientific, Dreieich, Germany). Flow curves for emulsions (without pectin) were performed with a sand-blasted coaxial cylinder Z-20. Emulgels studied were measured using a serrated plate–plate sensor (60 mm diameter; 1 mm gap). The rheological characterization for emulgels involved stress and frequency sweeps in small amplitude oscillatory shear experiments (SAOSs) and flow curves. Equilibration time prior to rheological tests was 5 min. All measurements were carried out at 25 °C.

### 2.5. Analysis of Physical Stability

Multiple light-scattering measurements were conducted, as a function of aging time, using Turbiscan Lab Expert (Formulaction, Toulouse, France) for the monitoring of the physical stability of all studied samples at 25 °C.

## 3. Results and Discussion

### 3.1. Exploring the Optimal Phycocyanin Concentration

**Figure 1:**
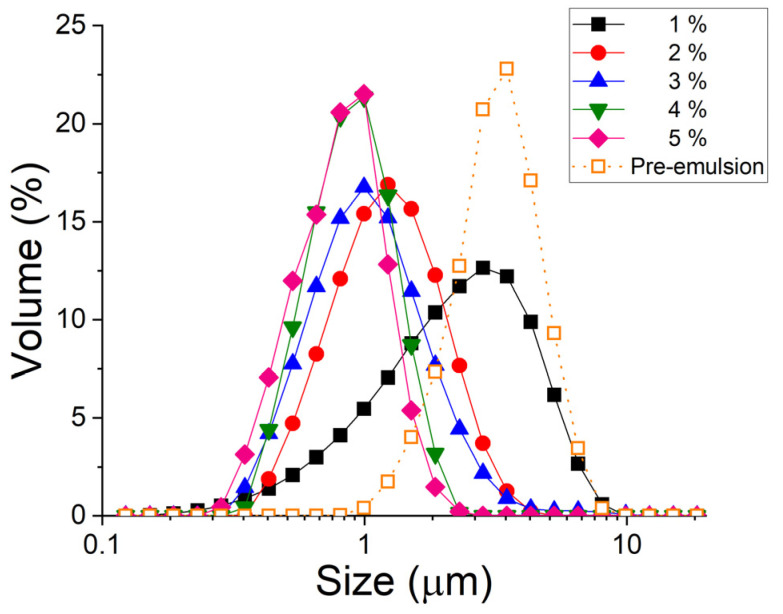
Droplet size distributions (DSDs) as a function of PC concentration for the emulsions studied. Aging time, 0 h.

Figure 1 shows the droplet size distributions (DSDs) for emulsions based on a mixture of avocado and lemon oils as a function of phycocyanin (PC) concentration. As expected, droplet size was reduced by an increase in PC concentration. This indicates that there was not enough emulsifier content, as it is widely known that the emulsifier concentration determines the size limit that can be reached in droplet size [13]. This fact can be understood by taking into account the fact that the emulsifier forms a layer at the interface, and if there is not enough emulsifier to cover the surface of all the droplets, they will coalesce to form new larger droplets until the point of full coverage is reached. A significant difference in span should be noted for the pre-emulsion (3 wt.% PC) and the 1% emulsion (1.243 and 1.696, respectively), which is also related to a clear lack of emulsifier to cover all the new interfaces created during the high-pressure emulsification process [14]. In addition, the Sauter and volumetric diameters (Table 1) underwent substantial changes in the pre-emulsion and microfluidized emulsion containing 3 wt.% of phycocyanin as a result of the high-pressure emulsification process.

As a conclusion from both Figure 1 and Table 1, it can be said that the best results in terms of the smallest sizes and lowest polydispersity are those obtained for the emulsion containing 5 wt.% PC. Similar results have recently been obtained with other emulsifying agents derived from sustainable biological resources, such as avocado phospholipids [15].

**Figure 2:**
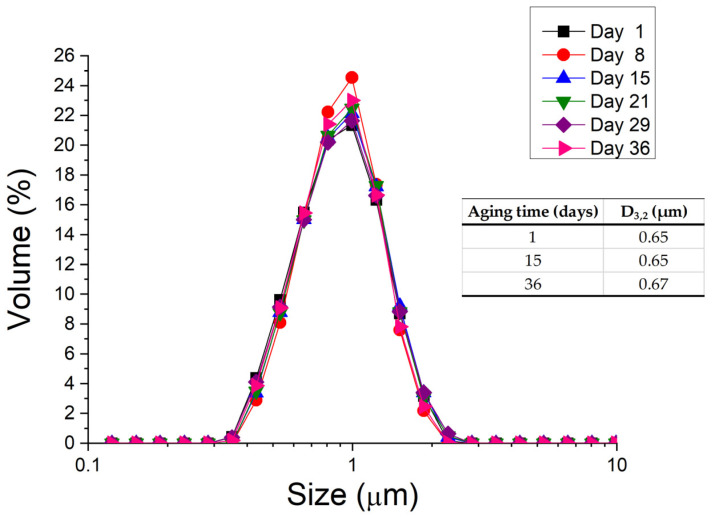
Droplet size distributions for the emulsion prepared with 5 wt.% of PC as a function of aging time.

In a subsequent step, the influence of aging time on the DSD for the emulsion containing 5 wt.% PC was studied. Figure 2 shows DSD for the emulsion containing 5 wt.% of PC as a function of aging time. It can be clearly observed that there are no significant differences in DSD for the aging times studied (see inset Figure 2), which reveals that it is possible to obtain at least a stable emulsion against coalesce/Ostwald ripening with this specific concentration of emulsifier. This is because only coalescence and Ostwald ripening destabilization phenomena could be detected with the evolution of DSD, unlike the creaming mechanism.

**Figure 3:**
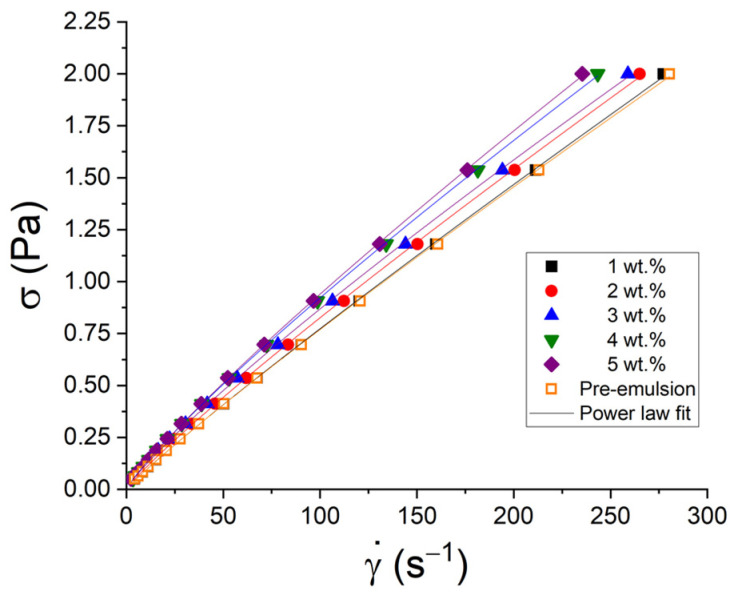
Flow curves as a function of PC concentration for the emulsions without pectin. Lines are the best fit to the power law model with the rheological parameters given in Table 2.

Figure 3 shows the flow curves for the emulsions studied as a function of PC concentration. It can be observed that the shear rate decreases with the concentration of phycocyanin at a fixed shear stress, which implies higher values of apparent viscosity. This can be clearly seen in Table 2, where the viscosity at 2 Pa is shown by way of example. The increase in viscosity is related to the smaller droplet sizes of the emulsions as a consequence of the role of the emulsifier. It can also be attributed to an increase in the viscosity of the continuous phase due to the presence of PC within it, at higher concentrations [16]. In addition, Table 3 shows the flow index and shear stress at 1 s^−1^, which is related to the flow consistency coefficient (k), and its value can be viewed as the value of apparent viscosity at the shear rate of unity. It was preferred to show shear stress at 1 s^−1^, taking into account that one of the limitations of the power law model is the dependence of the dimensions of k on the numerical value of n, which is why the values of k should not be compared when the values of n are different [17]. The flow index is indicative of the deviation from the Newtonian behavior of the fluid, that is, of its pseudoplastic character, and this is usually related to molecular interactions and the spatial ordering of the macromolecules between the layers of the fluid where this spatial ordering may or may not favor the motion of the fluid. The flow consistency coefficient, k, or in this case shear stress at 1 s^−1^, can be interpreted as a measure of its resistance to deformation [17]. Thus, a clear trend towards a decrease in the flow index with the concentration of phycocyanin (greater pseudoplastic character) is demonstrated. The pseudoplastic nature of these emulsions can be associated with slight flocculation of the samples due to the presence of PC in the continuous phase since the concentration of the oil phase is low (20 wt.%), which gives rise to weakly flocculated emulsions [18]. The pre-emulsion with 3 wt.% PC does not follow the trend of the group of systems studied because the concentration variable must also be considered. Therefore, for the same concentration (3 wt.%), n decreases and shear stress at 1 s^−1^ increases after processing (high pressure) to obtain the emulsion. This demonstrates that processing and its result, the occurrence of sub-micron and nanodroplets, causes a greater pseudoplastic character and greater resistance to deformation or, what is the same, greater viscosity at a given shear rate.

**Figure 4:**
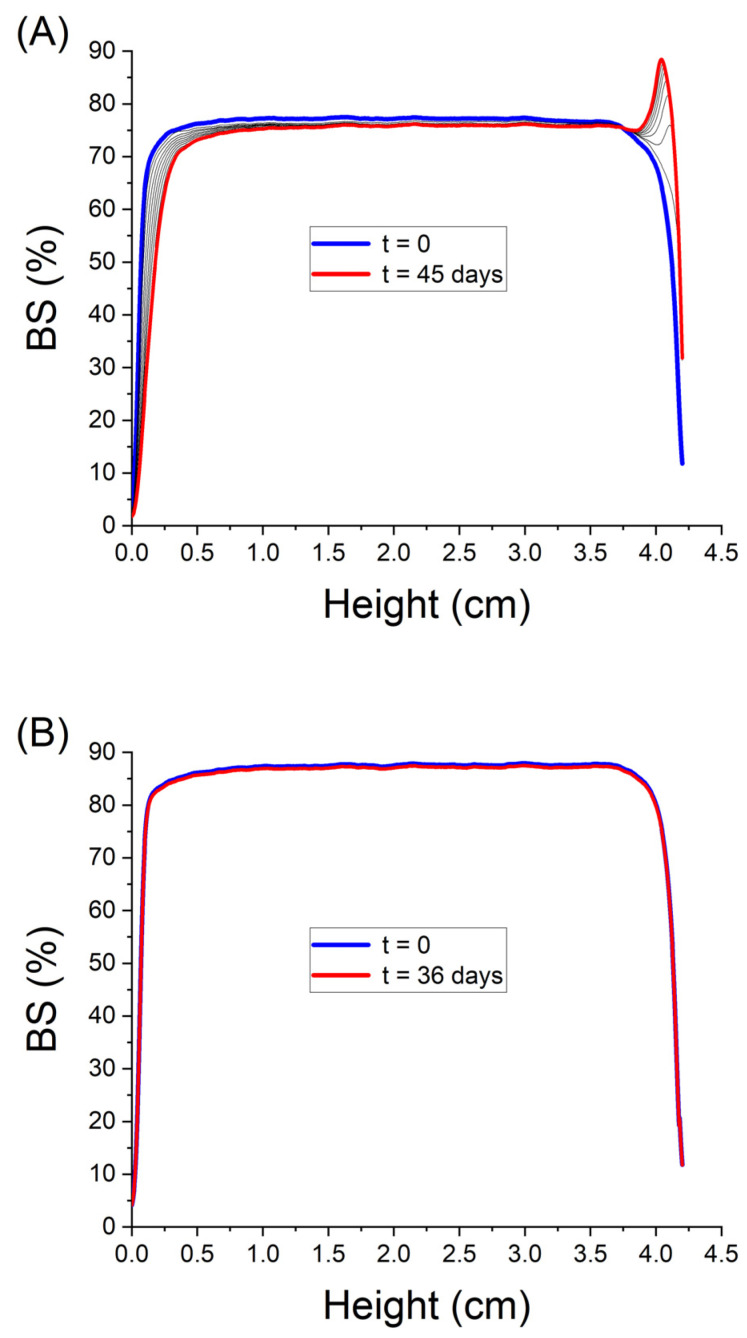
Backscattering versus container height as a function of time for (**A**) the emulsion formulated with 3 wt.% of PC and no pectin and (**B**) the emulgel formulated with 5 wt.% PC and 2.5 wt.% pectin. Temperature: 20 °C.

Figure 4A shows the variation in backscattering (BS) as a function of time and the height of the sample for the microfluidized emulsion containing 3 wt.% PC. A clear decrease in BS can be observed with time in the lower part of the measurement cell, indicating a creaming destabilization mechanism. In addition, the increase in BS over time in the intermediate zone of the vial can be associated with a flocculation process, as it was previously verified that there was no increase in droplet size at these times as a consequence of coalescence or Oswald ripening (see Figure 2).

**Figure 5:**
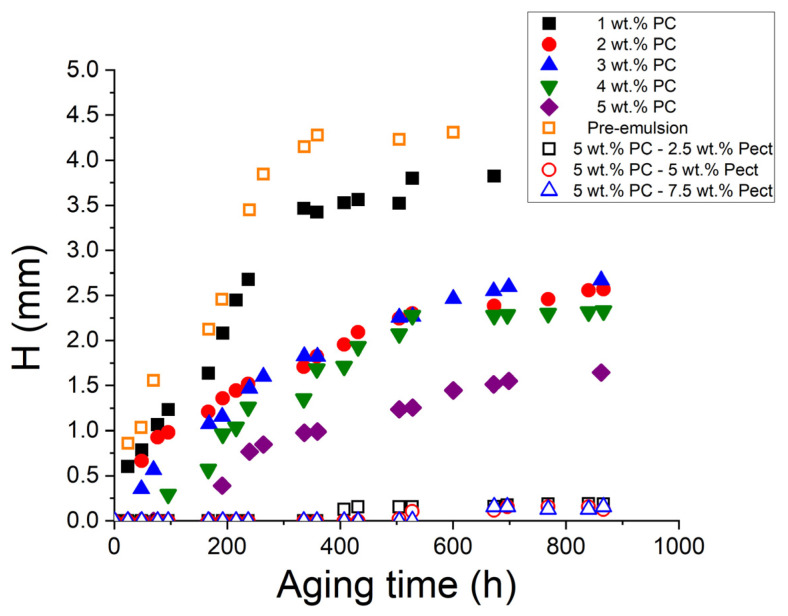
Creaming height as a function of the aging time for all studied emulsions. Samples kept under storage at 20 °C.

Focusing on the destabilization process by creaming, Figure 5 shows the height of creaming (H) versus the aging time for all the emulsions studied. Fitting of the results led to a delay time for creaming (t_0_) and the creaming rate (CR), which was determined as the slope of the line. It should be noted that the pre-emulsion containing 3 wt.% and the 1 wt.% emulsion show similar behavior, with the delay time of creaming equal to zero and a high and similar initial creaming rate until reaching a maximum value of H, which indicates total phase separation. This occurs for different reasons in both emulsions: in the case of the pre-emulsion, it is due to the lack of processing and of nano size, and in the case of the 1% emulsion, it is due to the lack of an emulsifier. As for the rest of the emulsions studied, it should be noted that while, in the 2–4 wt.% range of PC, creaming occurred at shorter aging times, the 5 wt.% emulsion showed a much longer delay time for creaming. Regarding the creaming rate, it can be considered that they are significantly similar for the most phycocyanin-concentrated systems. As expected, all these results have a direct relationship with the distribution and size of droplets, such that the smaller the mean diameters, the greater the stability against creaming [19]. The destabilization mechanism is present in all the studied systems due to the low viscosity of the samples. For this reason, and as a strategy to inhibit the destabilization process, a subsequent study on the influence on the stability of these emulsions via the addition of a stabilizer (citrus peel pectin) to the emulsion with the lowest droplet mean diameter and higher stability (5 wt.% PC) is proposed.

### 3.2. Strategy to Solve the Creaming Destabilization Process

**Figure 6:**
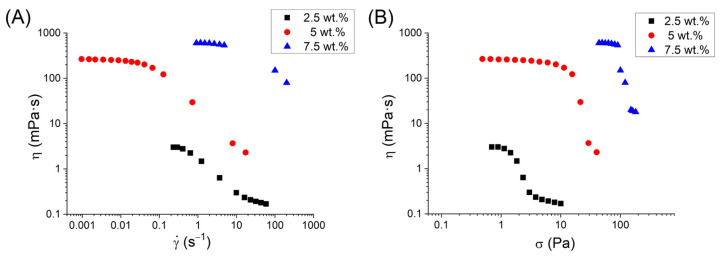
Viscosity versus (**A**) shear rate and (**B**) shear stress for the systems based on PC as a function of citrus peel pectin concentration.

In order to enhance the physical stability of the systems, pectin was incorporated at 2.5, 5, and 7.5 wt.% into the emulsion formulated with 5 wt.% of phycocyanin. Figure 6A shows the flow curves for PC-based emulsions formulated with citrus peel pectin as a function of pectin concentration. All systems showed a decrease in apparent viscosity with the shear rate. A tendency to reach a plateau in viscosity at very low shear rates can also be observed. Hence, these emulgels present very shear-thinning behavior with the occurrence of zero-shear viscosity. The latter is related to the stability of the emulsions against creaming [20]. The very shear-thinning behavior is characterized by a lack of data in a certain range of shear rates, which is due to a marked increase in the shear rate in a narrow shear stress range. This is related to the appearance of yield stress [21]. In the apparent viscosity–shear stress plot (Figure 6B), an abrupt decrease in viscosity is shown at a certain shear stress. The yield stress is higher with increasing pectin concentration, as expected. Higher values of yield stress are related to the enhancer stabilities [22,23]. The yield stress values are shown in Table 3.

**Figure 7:**
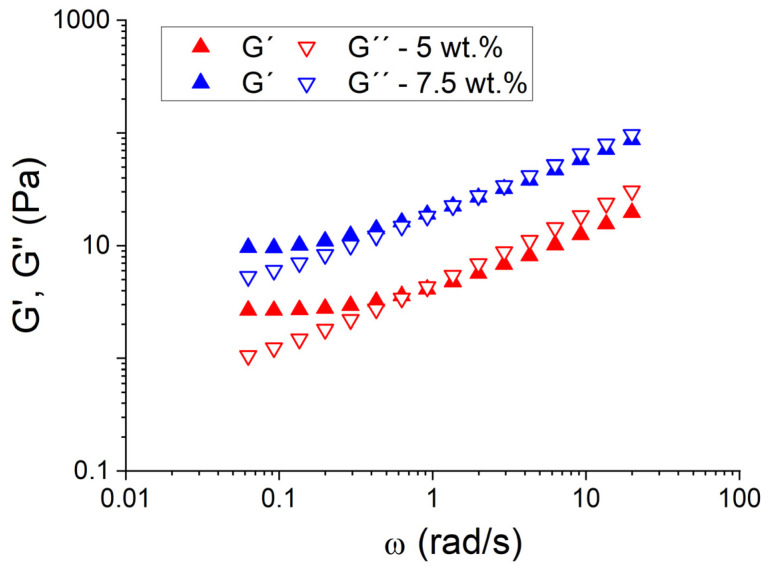
Mechanical spectra for the systems based on PC as a function of citrus peel pectin concentration.

Figure 7 shows the mechanical spectra for emulgels formulated with 5 and 7.5 wt.% of pectin. It is important to note that the data for emulsion containing 2.5 wt.% are not shown. This case was quite enlightening since both G′ and G″ fell throughout the whole shear stress range applied. This means that the response of 2.5 wt.% pectin was probably outside of the LVR at the lowest stress allowed by the rheometer.

The two most concentrated systems presented the same tendencies in viscoelastic moduli: G′ (elastic modulus) is higher than G″ (viscous modulus) in the lower frequency regime, and G′ is lower than G″ in the higher frequency regime because of a crossover point between G′ and G″ at lower frequencies. At the lower frequencies, the plateau window of the mechanical spectra, where the slopes of G’ at lower frequencies were close to zero, can be observed. This indicates the presence of an incipient weak gel, which seems to indicate improved physical stabilities against creaming (see Figure 5) for the most pectin-concentrated emulsions and especially that with higher values of G′ and G″ (emulsion containing 7.5 wt.% of pectin). In addition, at higher frequencies, the results obtained corresponded to part of the transition zone where G″ grows faster [24].

## 4. Conclusions

The preliminary studies for determining the optimal PC concentration revealed that the best results in terms of the smallest sizes and lowest polydispersity were those obtained for the emulsion containing 5 wt.% PC. Also, significant differences in the DSD were not detected for 36 days of aging time for this concentration, which demonstrates that at least this emulsion is stable against coalesce/Ostwald ripening as a consequence of the emulsifier forming a layer that recovers and protects the interface. A previous study also revealed that all emulsions formulated with different concentrations of PC showed pseudoplastic behavior, with an increase in flow index with PC concentration. This fact indicates a slight induced flocculation due to PC concentration, which could result in an increase in the viscosity of the emulsion and, hence, favors the physical stability against creaming. These results could also be proved by MLS, which confirmed the occurrence of creaming and slight flocculation. Despite the fact that a creaming destabilization process was detected for all PC concentrations studied due to the low viscosity of the samples, the 5 wt.% emulsion showed a much longer delay time for the destabilization process. In order to enhance physical stability, different pectin concentrations (2.5, 5, and 7.5 wt.%) were incorporated into the systems developed to form a gel in the continuous phase that increases its viscosity in order to retard creaming. The flow curves of the emulgels formed revealed a very shear-thinning behavior identified by a marked decrease in viscosity in a narrow shear rate range, which is associated with a yield point and a tendency to reach a zero-shear viscosity value in the low shear-rate region. Both zero-shear viscosity and yield stress may be related to the stability of the emulsions against creaming, with the most stable emulsions being those with higher zero-shear viscosity and yield point. The emulsion formulated with the lowest pectin concentration (2.5 wt.%) did not show a significant linear viscoelastic range since the response was probably outside of the LVR at the lowest stress allowed by the rheometer. However, the most concentrated emulsions showed measurable viscoelastic behavior, revealing a mechanical spectrum that corresponds to the plateau zone and the onset of the transition zone. The occurrence of an incipient weak gel can be justified by the fact that the slopes of G′ at lower frequencies were close to zero. This can be related to the decrease in the creaming rate, which was also demonstrated by MLS. As our understanding of phycocyanin continues to expand, this work can contribute to various fields, from health and medicine to food technology and beyond.

## Figures and Tables

**Table 1:** Sauter mean diameters (Ds_3,2_), volumetric mean diameter (D_4,3_), and span as a function of PC concentration for the emulsions studied. Aging time, 0 h. The approximate percentage of variability in the technique with the experimental protocol and type of emulsions is 5% for D_3,2_, 8% for D_4,3_, and 8% for the span.

Phycocyanin Concentration (wt.%)	D_3,2_ (μm)	D_4,3_ (μm)	Span
1	1.43	2.31	1.696
2	0.98	1.21	1.239
3	0.83	1.08	1.115
4	0.75	0.85	0.929
5	0.65	0.77	0.887
Pre-emulsion (3 wt.%)	1.69	2.40	1.243

**Table 2:** Influence of PC concentration on the fitting parameters to the power law model. Aging time, 0 h. The approximate percentage of variability in the technique with the experimental protocol and type of emulsions is 5% for all parameters.

Phycocyanin Concentration (wt.%)	σ_1_ (Pa)	n	η_2Pa_ (mPa·s)
1	0.011	0.92	7.21
2	0.013	0.90	7.54
3	0.016	0.86	7.72
4	0.017	0.86	8.21
5	0.017	0.85	8.49
Pre-emulsion (3 wt.%)	0.011	0.89	7.13

**Table 3:** Influence of citrus peel pectin concentration on the yield stress values. Aging time, 0 h. The approximate percentage of variability in the technique with the experimental protocol and type of samples is 5% for yield stress.

Pectin Concentration (wt.%)	Yield Stress (Pa)
2.5	1.8
5	20
7.5	125

## Data Availability

The data presented in this study are available on request from the corresponding author.
